# 

**Published:** 1921-03-19

**Authors:** 


### Forthcoming Events.

Hospital Saturday Fund.—Forty-seventh annual meeting, Saturday, April 9, at 3 p.m., at the Mansion House. Addresses will be given by Sir William Collins, K.C.V.O., and Sir Philip Gibbs, K.B.E.

St. Peter's Hospital, Henrietta Street, Covent Garden, W.C.—Annual General Meeting at the hospital, Tuesday, March 22, at 5 p.m. A motion will be proposed to amend Rule XII. of the Regulations by adding the following words —*viz.*, "and shall have power to co-opt one or more additional members to act for the rest of the current year."

Great Northern Central Hospital, Holloway, N. 7.—The Annual Council of Governors, Tuesday, March 22, 4.30 p.m., in the Board Room. The Marquis of Northampton (Chairman) will preside.

### Gifts and Bequests.

Mr. J. D. A. Norris, of the Devonshire Club, has bequeathed £100 to the Lock Hospital.

The Rev. Hugh Holland-Howard, for forty years rector of Melverley, Salop, has left £100 to the Shropshire Eye, Ear and Throat Hospital.

A memorial bed has been founded at the York County Hospital out of the funds of the York V.A.D. and St. John Ambulance Association.

Mrs. Drake has given the sum of £1,000 for the provision of a ward in Nakuru Hospital, Kenya Colony, to be called the "Drake Ward," in memory of her son, who was killed in the East African campaign.

St. Mary's Hospital, Manchester, has received £1,000 from Mrs. E. Lees for the endowment of a bed, and £750 from the Anson Golf Club for the endowment of a cot.

The King Edward VII. Hospital, Cardiff, has received £1,050 from Messrs. Will and Jack Gibson, well known in local athletic circles, for the endowment of a bed in memory of their late father. As a token of gratitude an ex-patient, who remains anonymous, has contributed £1,050 to this hospital for the endowment of a bed. A cheque for £50 has also been received by the hospital from Colonel Anthony, with an intimation that he hopes to be able to give £100 a year for the salary of the sister-in-charge of Anthony House.

Ipswich and East Suffolk Hospital receives £100 under the will of Mr. W. G. Synnot, solicitor, of Ipswich.

The Royal Victoria and West Hants Hospital will receive £200 bequeathed by Mr. Sydney R. J. White, of Bournemouth.

The ex-Service men of Clewer and Dedworth have given their United Services Fund Christmas gift of 5s. each (amounting in all to £98 10s.) to the King Edward VII. Hospital, Windsor.

Amongst the grants from the National Relief Fund are £7,000 to the Royal Portsmouth Hospital, £12,500 to the Bath Royal United Hospital, £5,400 to Wolverhampton General Hospital, and £4,400 to Mansfield Hospital.

Miss Elizabeth Woods, of Lancaster, has left £1,000 each to the Lancaster Infirmary and the Royal Asylum for Idiots, and £500 each to the Lancaster District Nursing Association and the Invalid Children's Association.

The Hospital of Saint Cross, Rugby, benefits to a considerable extent under the will of Mr. W. W. Shillitoe, a former builder, of Rugby. After making provision for a few small private legacies, Mr. Shillitoe directed that the whole of his property—which is estimated to be worth some thousands of pounds—shall be sold and the proceeds used to form a trust for the provision and maintenance of beds, to be known as the "Shillitoe" beds, at the hospital.

### Personalia.

Mrs. H. A. Richards, M.A., B.Ch., Cantab., M.R.C.S., L.R.C.P., has been appointed junior anæsthetist and assistant instructor in anæsthetics to King's College Hospital.

Dr. Robert A. Lyster, County Medical Officer for Hampshire, and Editor of *Public Health*, has been appointed a member of the reconstituted Central Midwives Board on the nomination of the Society of Medical Officers of Health.

Mr. Alan Andreas Cockayne, M.R.C.S., L.R.C.P., has been appointed assistant school medical officer and medical inspector of schools under the Newport (Mon.) Education Committee. Mr. Cockayne, who graduated at Cambridge, was, from 1917 to 1919, temporary surgeon in the Navy, and subsequently studied public health questions.

Mr. Herbert Gill and Mr. S. Ingham, Chairman and Vice-Chairman respectively of the Bradford Hospital Fund, have been appointed Vice-Presidents of the Bradford Royal Infirmary as a mark of appreciation of the Fund's efforts.

Dr. W. H. Johnston, M.D., F.R.C.S. Edin., has been appointed medical superintendent of the Orthopædic Centre, Birmingham, comprising the hospitals at Highbury, Uffculme, and Sorrento. Dr. Johnston at present holds the office of Deputy Commissioner Medical Services (Ministry of Pensions) for Orthopædics, West Midlands Region. During the war he gained considerable experience in orthopædic surgery with the Forces.

Mr. Stanley E. Wilkins, who retired only last year from the secretaryship of the Royal Bucks Hospital, has now been elected a life governor in recognition of his services during the fourteen years when he held this post.

Mr. Paul Waterhouse and Mr. George Hornblower have been appointed by the Committee of University College Hospitals as joint architects in connection with the reconstruction scheme which is largely the outcome of the magnificent gift of the Rockefeller Foundation. Mr. Waterhouse collaborated with his father, the late Alfred Waterhouse, R.A., in the design of the present hospital buildings, the funds for which were in a large measure provided by the late Sir Blundell Maple, and he brought the scheme to completion after his father's death, and was the sole architect of the Medical School. Mr. Hornblower has been for many years the consulting architect of the Hospital Committee.

### Matrons' and Sisters' Appointments.

Market Harborough Union Infirmary.—Miss Lucy Stanley has been appointed head nurse. She was trained at the Erdington Infirmary, Birmingham, has been head nurse at the Chipping Norton Union, and superintendent nurse of the Poole and Isle of Thanet Unions.

Plymouth Mental Hospital.—Miss G. F. Burnell has been appointed matron. She was trained at West London Hospital, has been charge nurse at the City Hospital, Birmingham, senior staff nurse at the Statutory Hospital, Bath, night superintendent at the City of Birmingham Fever Hospital, assistant matron at the Cheddleton Asylum, Staffs, and has seen four years' war service in France.

Beckett Street Infirmary, Leeds.—Miss Hannah Josephine Harkin has been appointed matron. She was trained at the Parish of St. Pancras Hospital, has been superintendent nurse at the Hartlepool and Bromley Unions, assistant matron of the Edinburgh War Hospital, superintendent nurse of the Coventry Union, and matron of Birkenhead Union.

Hopital du Deux Alice Uccle, Brussels.—Miss Kathleen Bell has been appointed matron. She was trained at the Norfolk and Norwich Hospital, and in her fourth year served on the private staff, following this by surgical nursing in a Home. She then went as staff nurse to the Cancer Hospital, Fulham Road, thence as sister to the Dreadnought Hospital, working successively in the tubercular wards, as home sister, night superintendent, and sister of the senior surgical floor. Miss Bell is now taking a six-months' housekeeping course at her hospital.

Porth District Hospital.—Miss F. M. Pratt has been appointed matron. She was trained at King Edward VII. Hospital, Cardiff, and has been sister at the Porth and District Hospital.

Saunderton Sanatorium.—Miss Bowden has been appointed matron. She was trained at Marylebone Infirmary and at the Royal National Hospital for Consumption, Ventnor. She has since been charge nurse at the Frank James Memorial Hospital, Cowes, ward sister and temporary home sister at Selly Oak Hospital, Birmingham, night sister at the Royal National Hospital for Consumption, Newcastle, Co. Wicklow, night superintendent at the Infirmary, Acton Lane, Middlesex, home sister at Queen Mary's Hospital for Children, Carshalton, matron of the Mitchell Memorial Sanatorium, Leeds, sister at the Marylebone Infirmary and the Papworth Tuberculosis Colony. She is a member of the College of Nursing.

Union Hospital, East Dulwich Grove.—Miss Ethel Louisa Butcher has been appointed night superintendent. She was trained at Woolwich Infirmary, has been home sister at the South-Eastern Hospital for Children, Sydenham, and ward sister at the Mile End Military Hospital.

Aberdare and District General Hospital.—Miss A. Price has been appointed night sister. She was trained at the Royal Gwent Hospital, Newport.

